# Effect of Fluoride Exposure on the Mechanical Properties of NiTi Orthodontic Archwires: An In Vitro Study

**DOI:** 10.7759/cureus.107558

**Published:** 2026-04-22

**Authors:** Vilius Kosys, Gabija Streimikyte, Mantas Sidlauskas

**Affiliations:** 1 Odontology, Lithuanian University of Health Sciences, Kaunas, LTU; 2 Orthodontics, Lithuanian University of Health Sciences, Kaunas, LTU

**Keywords:** artificial saliva, in vitro study, orthodontic archwires, sodium fluoride solution, mechanical properties

## Abstract

Background

Fluoride-containing mouth rinses are widely used in orthodontic patients to prevent enamel demineralization. However, fluoride exposure may influence the mechanical properties of nickel-titanium (NiTi) orthodontic archwires, potentially affecting their clinical performance. This study aimed to evaluate the effect of a 905 ppm fluoride-containing mouth rinse on the mechanical properties of NiTi orthodontic archwires.

Methodology

An in vitro experimental study was conducted using 35 NiTi orthodontic archwires (0.014″), divided into experimental, control, and baseline groups. The experimental group was exposed to a fluoride-containing mouth rinse (905 ppm fluoride, corresponding to approximately 0.2% sodium fluoride) for 1, 1.5, and 2 hours, followed by storage in artificial saliva for 10, 15, and 20 days. Mechanical properties were assessed using a universal tension-compression testing machine. Statistical analysis was performed using the Mann-Whitney U test, with significance set at p-values <0.05.

Results

No statistically significant differences were observed in force at breakage between groups (p = 0.740). However, a statistically significant reduction in elasticity was identified following fluoride exposure, particularly with longer exposure duration and storage time.

Conclusions

Fluoride exposure did not affect the force at breakage of NiTi archwires but significantly reduced their elasticity. The effect increased with prolonged exposure, suggesting a cumulative influence of fluoride on the mechanical behavior of orthodontic archwires. Further in vivo studies are required to determine the clinical relevance of these findings.

## Introduction

Orthodontic treatment relies on the mechanical properties of nickel-titanium (NiTi) archwires, which are widely used for their superelasticity and shape-memory effect, enabling the delivery of relatively constant, light forces over a wide range of activation levels [1]. These properties are essential for delivering the continuous, controlled forces required for efficient tooth movement.

Fluoride-containing products are routinely recommended during orthodontic treatment as part of daily oral hygiene. Fluoride helps prevent enamel demineralization. However, previous studies have shown that fluoride exposure may influence the mechanical properties and corrosion behavior of orthodontic materials, particularly NiTi alloys [2,3].

The oral environment plays an important role in determining the behavior of orthodontic materials. Factors such as saliva composition (e.g., chloride ions and proteins), pH fluctuations (acidic conditions), and temperature variations may contribute to corrosion processes, including pitting and crevice corrosion, thereby increasing surface roughness, ion release, and altering the mechanical performance of archwires [4]. Acidic conditions (pH fluctuations), in particular, may enhance fluoride’s effects on NiTi alloys, potentially leading to degradation of the protective oxide layer and changes in elasticity and force delivery [3,5].

In addition to local factors, general health-related behaviors such as sleep may influence oral health outcomes; inadequate sleep has been associated with reduced salivary flow, impaired immune response, and an increased risk of periodontal disease, dental caries, and oral inflammation [6]. Sleep has been associated with general health outcomes, including impaired immune response and systemic inflammation, as well as oral health outcomes such as periodontal disease, dental caries, and oral inflammation [6]. However, its direct influence on the material properties of orthodontic materials remains unclear [7].

Oral hygiene practices, including the use of fluoride-containing mouth rinses, are commonly recommended during orthodontic treatment. Mouth rinsing is typically performed for approximately 60 seconds to ensure sufficient fluoride exposure and preventive efficacy. The duration and frequency of fluoride exposure may play a role in altering the mechanical properties of orthodontic archwires [8,9].

Given the widespread use of fluoride in orthodontic practice, it is important to evaluate its potential effects on the mechanical behavior of NiTi archwires. Fluoride-containing mouth rinses are widely used for routine oral hygiene and caries prevention in orthodontic patients [2]. Fluoride exposure has been shown to promote corrosion and alter the mechanical behavior of NiTi archwires [2]. Additionally, fluoride may reduce mechanical properties, potentially impairing tooth movement and prolonging orthodontic treatment [10,11].

This study aimed to evaluate the effect of a clinically relevant fluoride-containing mouth rinse (905 ppm fluoride), representative of commonly used over-the-counter products, on the mechanical properties of NiTi orthodontic archwires, specifically fracture load and elasticity.

## Materials and methods

**Figure 1:**
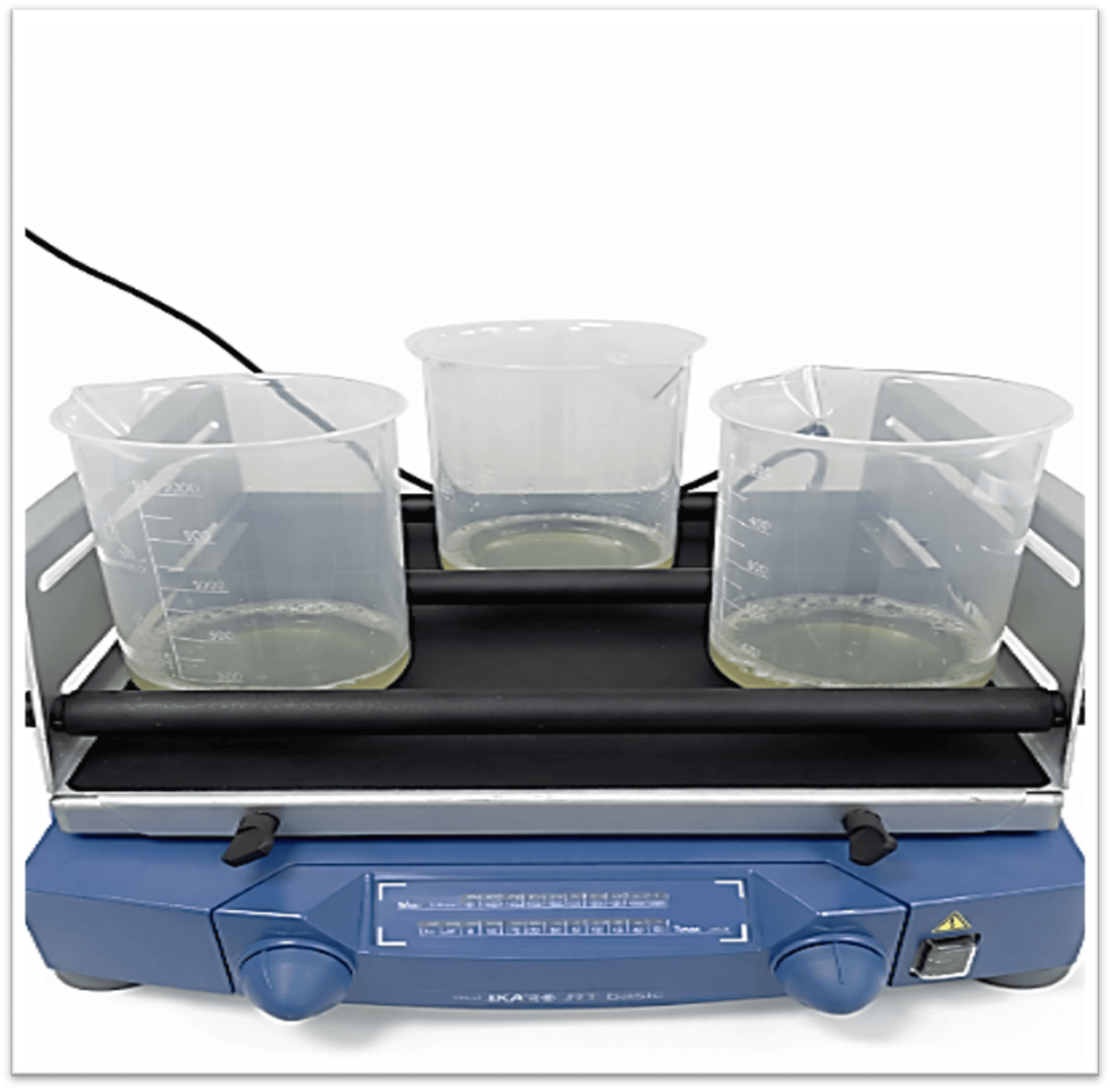
Experimental setup for fluoride exposure under magnetic stirring conditions. Source: Original figure created by the authors.

Specimens in the experimental group were immersed in a fluoride-containing mouth rinse (905 ppm fluoride) for 1, 1.5, and 2 hours under magnetic stirring to simulate dynamic clinical conditions. These immersion times were selected to approximate the cumulative effect of daily rinsing (twice per day for approximately 60 seconds) over clinical periods of 1, 1.5, and 2 months, respectively. A single-exposure protocol was used to standardize fluoride exposure and evaluate its prolonged effects during subsequent storage in artificial saliva. Following exposure, the samples were stored in artificial saliva for 10, 15, and 20 days to simulate short-term intraoral conditions. The experimental immersion setup under magnetic stirring is presented in Figure 1.

Specimens in the control group were stored in artificial saliva for the same time periods without fluoride exposure, while the baseline group was not exposed to any solution.

**Figure 2:**
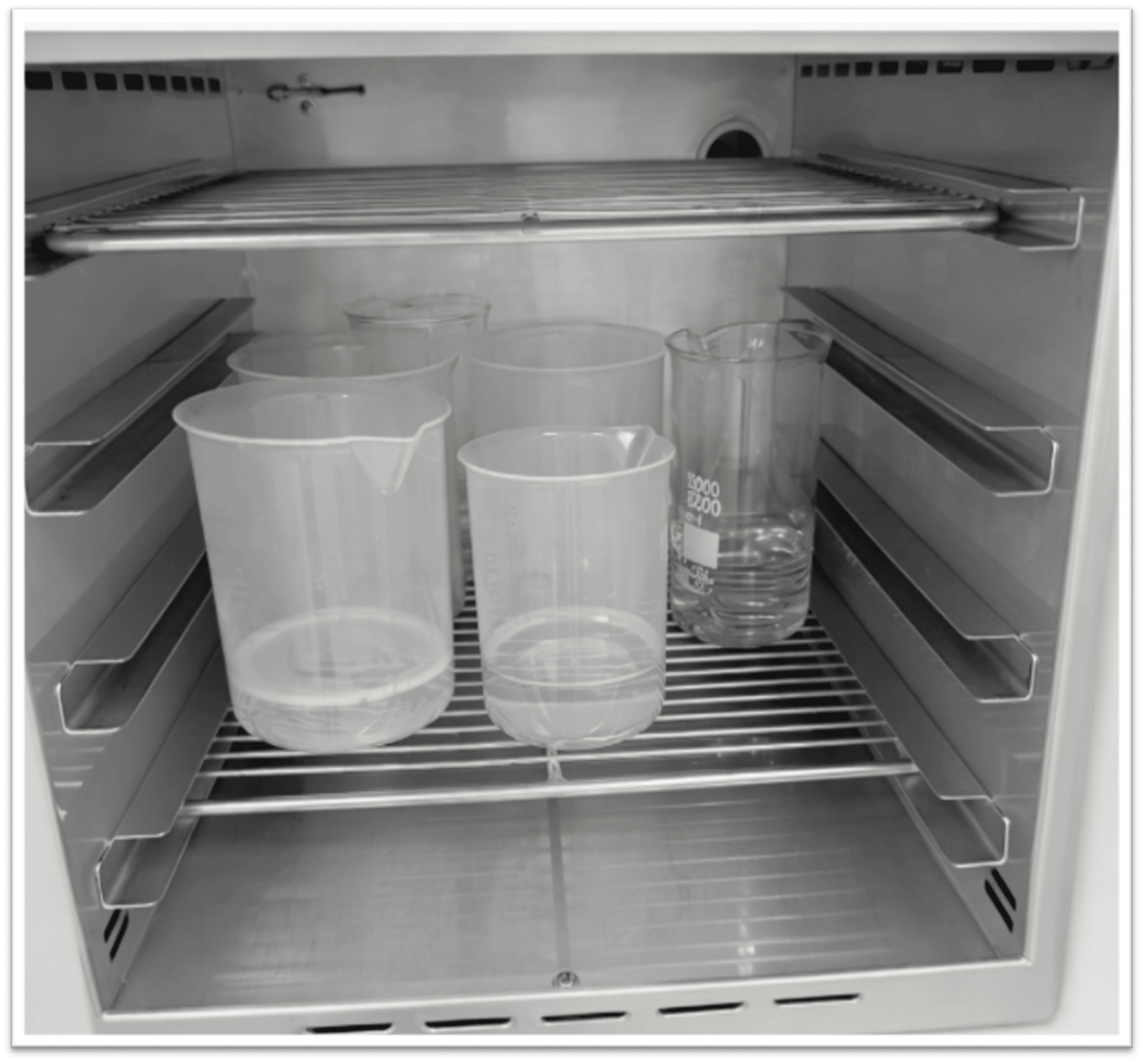
Storage of specimens in artificial saliva at 37°C to simulate intraoral conditions. Source: Original figure created by the authors.

All samples were maintained at a constant temperature of 37°C throughout the experiment. Storage conditions are illustrated in Figure 2.

Mechanical testing

**Figure 3:**
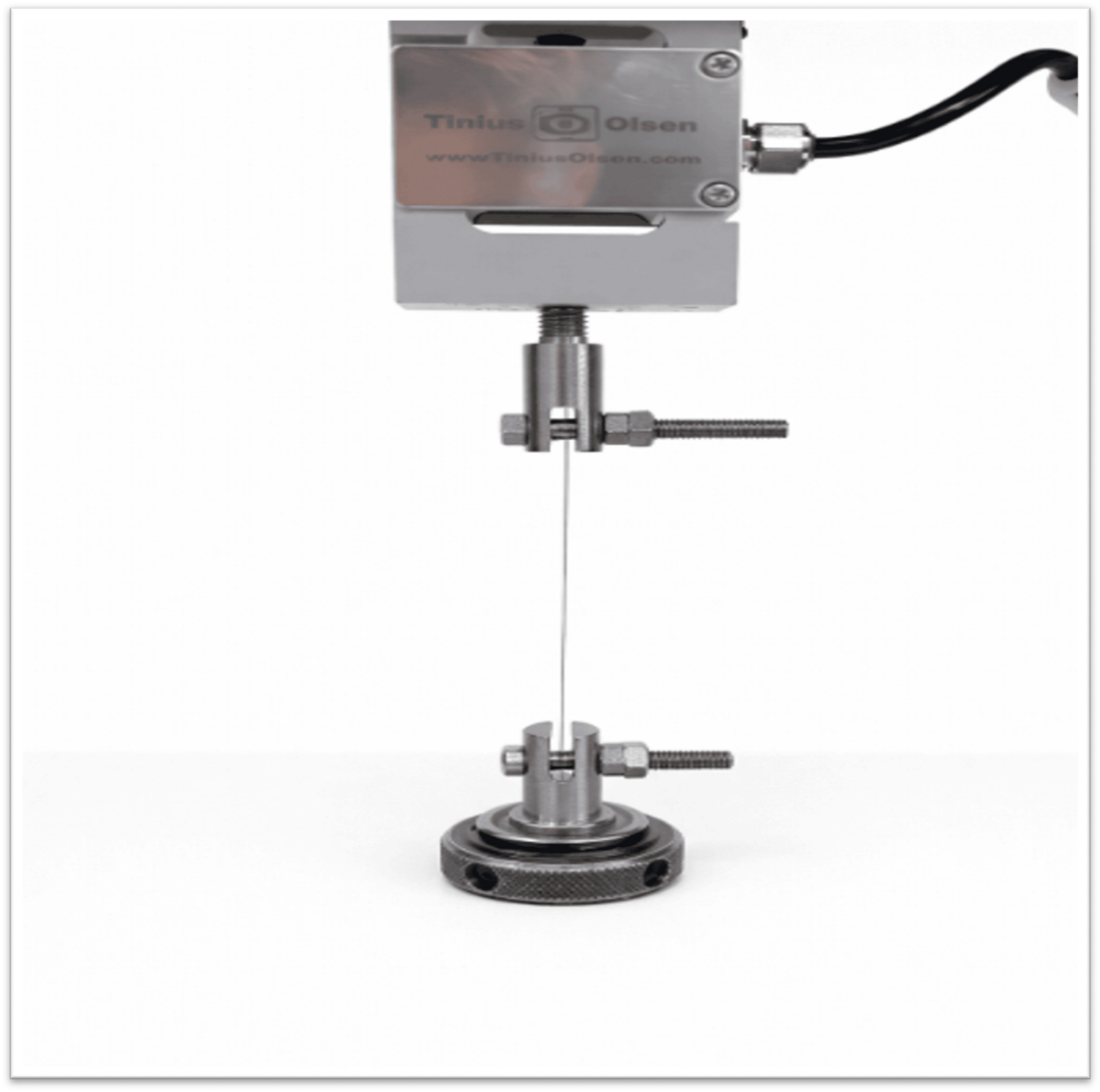
Mechanical testing of NiTi archwires using a universal tension-compression testing machine. Source: Original figure created by the authors.

Mechanical properties were evaluated using a universal tension-compression testing machine (10 kN) equipped with a 250 N load cell. The mechanical testing procedure is illustrated in Figure 3.

Statistical analysis

Statistical analysis was performed using SPSS Statistics version 29.0 (IBM Corp., Armonk, NY, USA). Differences between groups were assessed using the Mann-Whitney U test. A p-value <0.05 was considered statistically significant.

## Results

Experimental findings

**Figure 4:**
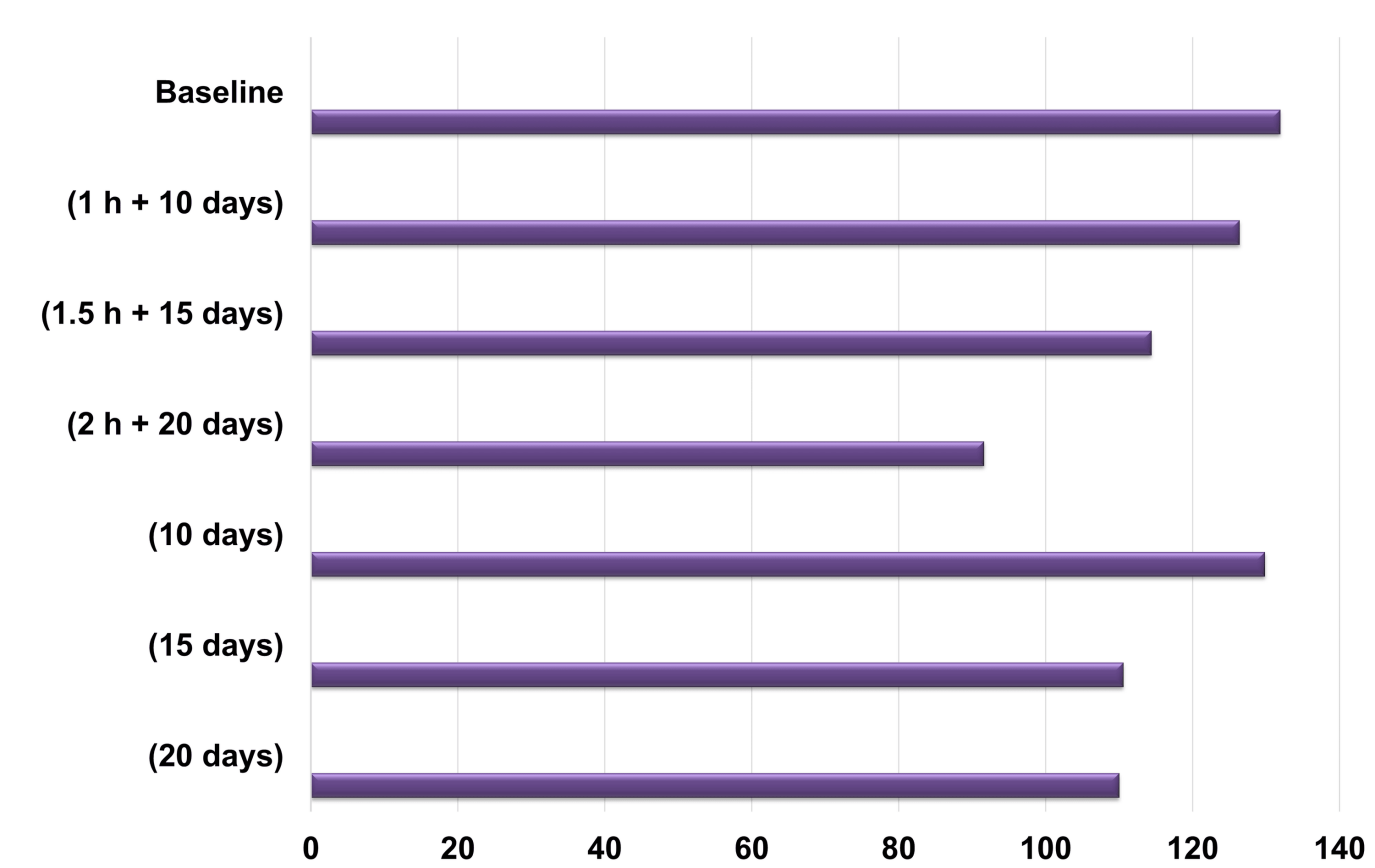
Changes in the fracture load of the archwires of 0.014" NiTi orthodontic archwires after fluoride exposure and storage in artificial saliva. Baseline: Unaffected group. 1 hour 10 days: Experimental group exposed to fluoride for 1 hour and artificial saliva for 10 days. 1.5 hours 15 days: Experimental group exposed to fluoride for 1.5 hours and artificial saliva for 15 days. 2 hours 20 days: Experimental group exposed to fluoride for 2 hours and artificial saliva for 20 days. 10 days: Control group exposed to artificial saliva for 10 days without fluoride. 15 days: Control group exposed to artificial saliva for 15 days without fluoride. 20 days: Control group exposed to artificial saliva for 20 days without fluoride.

The in vitro analysis demonstrated that exposure to a 905-ppm fluoride-containing mouth rinse altered the mechanical properties of NiTi orthodontic archwires. No statistically significant differences were observed in the fracture load of the archwires between the experimental and control groups (p = 0.740) (Figure 4).

The reduction in elasticity was more pronounced with increasing fluoride exposure, while storage in artificial saliva had a lesser effect, as summarized in Table 1.

**Table 1:** Changes in mechanical properties of NiTi archwires after fluoride exposure.

Group	Fluoride exposure	Storage time	Elasticity	Force at breakage
Experimental	905 ppm (1 hour)	10 days	Slight decrease	No significant change
Experimental	905 ppm (1.5 hours)	15 days	Moderate decrease	No significant change
Experimental	905 ppm (2 hours)	20 days	Marked decrease	No significant change
Control	No fluoride	10–20 days	No change	No change
Baseline	No exposure	-	Reference	Reference

**Figure 5:**
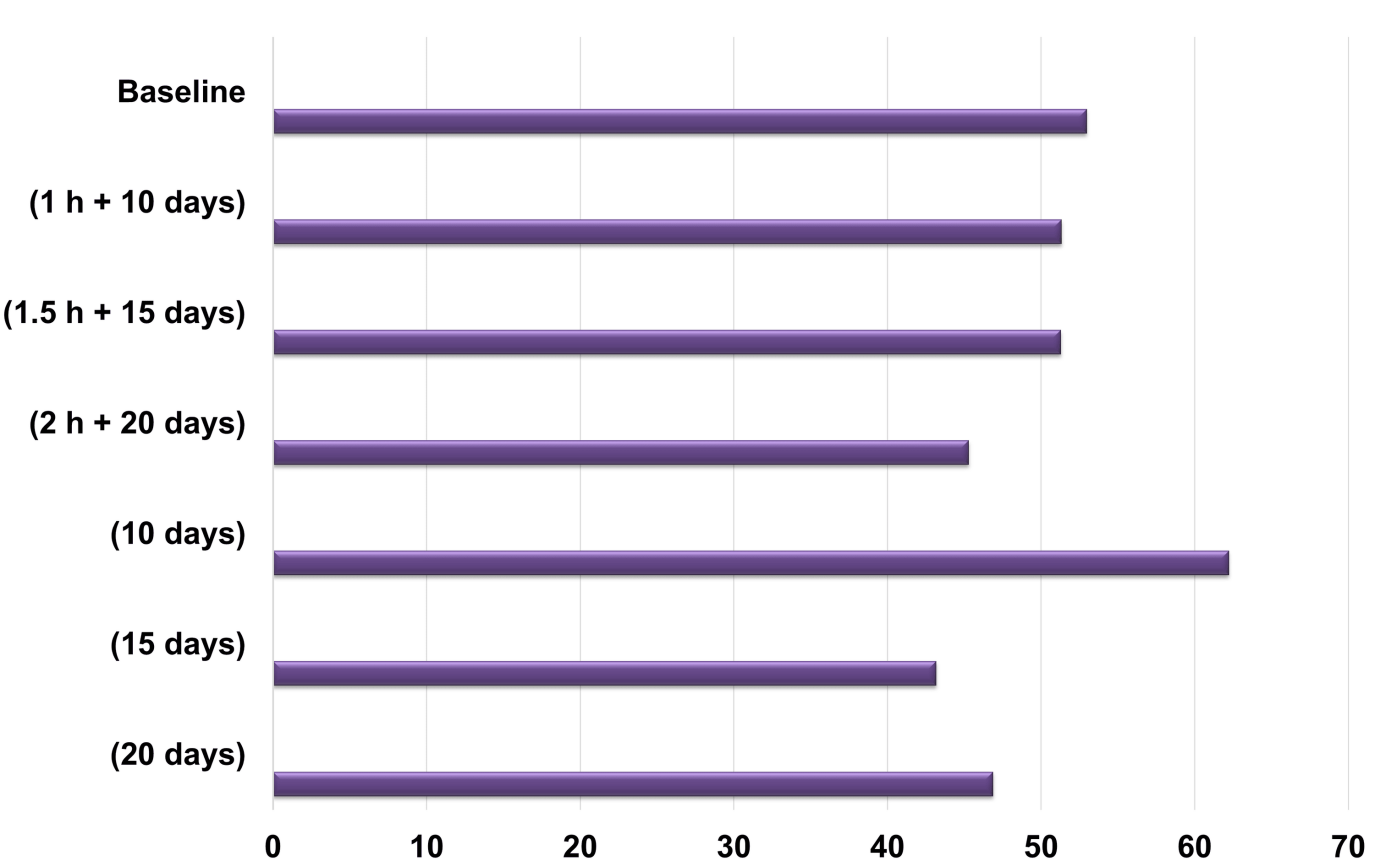
Changes in elasticity of 0.014" NiTi orthodontic archwires after fluoride exposure and storage in artificial saliva. Baseline: Unaffected group. 1 hour 10 days: Experimental group exposed to fluoride for 1 hour and artificial saliva for 10 days. 1.5 hours 15 days: Experimental group exposed to fluoride for 1.5 hours and artificial saliva for 15 days. 2 hours 20 days: Experimental group exposed to fluoride for 2 hours and artificial saliva for 20 days. 10 days: Control group exposed to artificial saliva for 10 days without fluoride. 15 days: Control group exposed to artificial saliva for 15 days without fluoride. 20 days: Control group exposed to artificial saliva for 20 days without fluoride.

The reduction in elasticity was classified based on the percentage decrease relative to baseline values: no change (<5%), slight (5-15%), moderate (15-30%), and marked (>30%). However, a statistically significant reduction in wire elasticity was identified following fluoride exposure. Archwires exposed to the fluoride mouth rinse and stored in artificial saliva demonstrated a significant reduction in elasticity compared with wires stored only in artificial saliva. The following significant reductions were noted: 1 hour fluoride exposure + 10 days storage, elasticity decreased to 51.4% (p = 0.047), and 2 hours fluoride exposure + 20 days storage, elasticity decreased to 45.3% (p = 0.016) compared with 10 days observed in artificial saliva (Figure 5).

Elasticity is a critical property of NiTi archwires, as it directly influences the delivery of continuous orthodontic forces. A reduction in elasticity may lead to altered unloading forces, less efficient tooth movement, and potential prolongation of treatment. These effects may be particularly relevant during the initial alignment phase, where thin NiTi wires are commonly used [9,10].

## Discussion

The present in vitro study evaluated the effect of fluoride exposure on the mechanical properties of NiTi orthodontic archwires. A fluoride concentration of 905 ppm was selected because it is commonly found in over-the-counter mouth rinses, thereby ensuring the clinical relevance of the experimental conditions. The results demonstrated that exposure to a 905 ppm fluoride-containing mouth rinse did not significantly affect the fracture load of the archwires. However, a significant reduction in elasticity was observed. It should be noted that the present findings are limited to a fluoride concentration of 905 ppm and therefore cannot be generalized to other concentrations or clinical exposure conditions.

These findings are consistent with previous studies, which report that fluoride exposure may compromise the mechanical behavior of NiTi alloys. It has been demonstrated that fluoride can reduce the mechanical properties of NiTi and CuNiTi wires, particularly affecting their elasticity and force delivery [9]. These findings are consistent with previous reports indicating that fluoride-containing agents may adversely affect the mechanical properties and force delivery of NiTi archwires [11]. In addition, fluoride exposure has been shown to alter the surface characteristics of NiTi wires, potentially increasing friction and affecting clinical performance [2].

The reduction in elasticity observed in the present study may be attributed to interactions between fluoride ions and the protective oxide layer of NiTi alloys. However, this mechanism was not directly evaluated in this study and is based on previously reported findings. Fluoride has been reported to disrupt the passive titanium oxide layer, potentially leading to increased corrosion and degradation of material properties [12,13]. Previous studies have shown that fluoride exposure, especially in acidic conditions, can significantly accelerate corrosion by disrupting the protective oxide layer of NiTi alloys [14]. In particular, acidic fluoride environments may accelerate corrosion and adversely affect the mechanical stability of NiTi wires [11]. Previous studies have demonstrated that acidic fluoride environments significantly reduce the corrosion resistance of NiTi archwires, thereby compromising their mechanical stability [15].

Corrosion and intraoral aging processes are known to significantly influence the performance and biocompatibility of orthodontic materials [2]. Furthermore, exposure to fluoride-containing solutions may increase the susceptibility of NiTi alloys to corrosion and ion release, thereby contributing to mechanical degradation [16].

An important observation in this study was that longer exposure duration and extended storage time resulted in greater changes in elasticity. This suggests that cumulative fluoride exposure may have a progressive effect on NiTi archwires. Prolonged exposure to fluoride solutions has been associated with increased surface degradation and reduced mechanical performance [11].

From a clinical perspective, fluoride-containing products remain essential for preventing enamel demineralization during orthodontic treatment [5,7]. Therefore, the potential negative effects on archwire properties should be interpreted cautiously. The benefits of fluoride use likely outweigh the risks. However, clinicians should be aware of possible material changes, particularly with prolonged exposure.

Furthermore, oral environmental factors such as saliva composition and pH fluctuations may enhance corrosion processes and interact with fluoride effects [17]. Therefore, these in vitro findings should be interpreted with caution when applied to clinical (in vivo) conditions. Additionally, as only a single fluoride concentration was evaluated, no conclusions regarding dose-response relationships can be drawn.

The limitations of this study include its in vitro design, which may not fully replicate the complexity of the oral environment. Factors such as salivary flow, pH variations, and mechanical stresses were not fully simulated. Future studies should include in vivo investigations and evaluate long-term clinical outcomes.

## Conclusions

Within the limitations of this in vitro study, including the absence of complex oral conditions (such as biofilm, temperature fluctuations, and mechanical loading) and the use of an accelerated exposure protocol, exposure to a 905 ppm fluoride-containing mouth rinse did not significantly affect the fracture load of NiTi orthodontic archwires. However, a statistically significant reduction in elasticity was observed, with the effect becoming more pronounced as fluoride exposure duration increased, suggesting a cumulative effect on the mechanical properties of the archwires. Although fluoride-containing products play an essential role in preventing enamel demineralization during orthodontic treatment, their potential influence on orthodontic materials should be considered. Further in vivo studies are needed to determine the clinical relevance of these findings.
